# BADERI: an online database to coordinate handsearching activities of controlled clinical trials for their potential inclusion in systematic reviews

**DOI:** 10.1186/s13063-017-2023-3

**Published:** 2017-06-13

**Authors:** Hector Pardo-Hernandez, Gerard Urrútia, Leticia A. Barajas-Nava, Diana Buitrago-Garcia, Julieth Vanessa Garzón, María José Martínez-Zapata, Xavier Bonfill, on behalf of the Iberoamerican Cochrane Network

**Affiliations:** 1 Iberoamerican Cochrane Centre, Sant Pau Biomedical Research Institute (IIB Sant Pau), Carrer Sant Antoni Maria Claret, 167, Pavelló 18, Ground Floor, Barcelona, Spain; 2 CIBER Epidemiología y Salud Pública (CIBERESP), Barcelona, Spain; 3 Evidence-Based Medicine Research Unit, Hospital Infantil de Mexico Federico Gómez (HIMFG), Health National Institute, Iberoamerican Cochrane Network, Mexico City, Mexico; 4 Division of Research, Fundación Universitaria de Ciencias de la Salud, Hospital San José/Hospital Infantil de San José, Bogotá, Colombia; 5 Facultad de Ciencias de la Salud Eugenio Espejo, Universidad Tecnológica Equinoccial, Quito, Ecuador; 6 Universitat Autònoma de Barcelona, Barcelona, Spain

**Keywords:** Information storage and retrieval, Database searching, Database development, Systematic reviews, Randomized controlled trial, Handsearching

## Abstract

**Background:**

Systematic reviews provide the best evidence on the effect of health care interventions. They rely on comprehensive access to the available scientific literature. Electronic search strategies alone may not suffice, requiring the implementation of a handsearching approach. We have developed a database to provide an Internet-based platform from which handsearching activities can be coordinated, including a procedure to streamline the submission of these references into CENTRAL, the Cochrane Collaboration Central Register of Controlled Trials.

**Methods:**

We developed a database and a descriptive analysis. Through brainstorming and discussion among stakeholders involved in handsearching projects, we designed a database that met identified needs that had to be addressed in order to ensure the viability of handsearching activities. Three handsearching teams pilot tested the proposed database. Once the final version of the database was approved, we proceeded to train the staff involved in handsearching.

**Results:**

The proposed database is called BADERI (Database of Iberoamerican Clinical Trials and Journals, by its initials in Spanish). BADERI was officially launched in October 2015, and it can be accessed at www.baderi.com/login.php free of cost. BADERI has an administration subsection, from which the roles of users are managed; a references subsection, where information associated to identified controlled clinical trials (CCTs) can be entered; a reports subsection, from which reports can be generated to track and analyse the results of handsearching activities; and a built-in free text search engine. BADERI allows all references to be exported in ProCite files that can be directly uploaded into CENTRAL. To date, 6284 references to CCTs have been uploaded to BADERI and sent to CENTRAL. The identified CCTs were published in a total of 420 journals related to 46 medical specialties. The year of publication ranged between 1957 and 2016.

**Conclusions:**

BADERI allows the efficient management of handsearching activities across different countries and institutions. References to all CCTs available in BADERI can be readily submitted to CENTRAL for their potential inclusion in systematic reviews.

**Electronic supplementary material:**

The online version of this article (doi:10.1186/s13063-017-2023-3) contains supplementary material, which is available to authorized users.

## Background

Systematic reviews and meta-analyses of randomized controlled trials (RCTs) provide the best evidence on the effect of health care interventions [1]. They review and integrate the available evidence through an assessment of research results, the methodological quality, and the risk of bias of the corresponding studies, facilitating an estimation of the confidence that can be placed on its conclusions [2], as proposed by the Grading of Recommendations Assessment, Development and Evaluation (GRADE) approach [3, 4]. Systematic reviews rely on a comprehensive and unbiased identification of available studies [5, 6]; developers must therefore be aware of the possibility of dissemination bias when conducting their literature searches. Dissemination bias has been defined as “the publication or non-publication of research findings, depending on the nature and direction of the results” [1].

Several initiatives around the world are currently committed to raising awareness and addressing the issue of dissemination bias, including AllTrials [7] and REWARD (Reduce research Waste And Reward Diligence) [8], as well as the Declaration of Helsinki [9], the World Health Organization’ (WHO’s) standards and operational guidance for ethics review of health-related research with human participants [10], the Code of Conduct of the Committee on Publication Ethics [11], and the ethical resources provided by the World Association of Medical Editors [12, 13], among others [14].

An approach to potentially address the issue of dissemination bias involves handsearching journals in order to identify controlled clinical trials (CCTs). Handsearching is defined as a progressive, page-by-page examination of all issues of a given journal, assessing all sections until each article can either be dismissed or classified as a CCT [15]. By implementing a handsearching strategy, issues of poor indexation and non-detection of studies published in journals not indexed in major databases or published in different languages can be overcome. Studies that have compared the proportion of studies identified via handsearching against those identified adopting electronic search strategies confirm the superiority of the handsearching approach [16–19].

One of the main promoters of handsearching worldwide is the Cochrane Collaboration, through different initiatives coordinated among review groups and Cochrane centres. As such, the Iberoamerican Cochrane Centre (IbCC), in collaboration with the Iberoamerican Cochrane Network (IbCN), conducts an initiative aimed at identifying all CCTs published in Spain and Latin America [20]. The project consists of handsearching journals of several medical specialties, obtaining the full text of any CCT that has been published, and carrying out a descriptive analysis of the main characteristics and potential risk of bias of the identified CCTs [17, 20, 21]. Through this effort, more than 4000 articles have been identified to date in more than 300 journals that have been handsearched completely or partially. Additionally, references to the CCTs identified are submitted to the Central Register of Controlled Trials (CENTRAL), the Cochrane Collaboration repository of CCTs [22, 23]. The results of these efforts have been disseminated in several publications, including studies on handsearching of CCTs in Dermatology [24, 25], Physiotherapy [26], Gynaecology [17], patient safety [16], General and Internal Medicine [27], Anaesthesiology (one journal in Spain) [28], and Dentistry (Villanueva J, Delgado I, Saldarriaga J, García Gargayo M, Amaro Y, Zapata S, Núñez L, Zamorano G, Pardo-Hernandez H, Bonfill, X, Martin C: Identification and description of controlled clinical trials in Spanish language dentistry journals, submitted), among others. Likewise, handsearching activities are underway again for Anaesthesiology, as well as for Geriatrics, Neurology, Oncology, Paediatrics, and Orthopaedics and Traumatology.

In order to address these challenges, we aimed to develop an Internet-based platform from which the handsearching activities could be coordinated. This tool would serve as a secure database for registering the journals that have been handsearched and the CCTs that have been identified. Additionally, it would facilitate coordinating the activities of several handsearching teams in different countries and institutions in Spain and Latin America, tracking the completed work to avoid duplication, verifying results, classifying and storing the CCTs identified, and planning future undertakings. Lastly, this platform would expedite the submission of the identified CCTs to CENTRAL for potential inclusion in systematic reviews and other documents of synthesis. In this article we present the methodology adopted to design and create this database and the results of its launching and implementation.

## Methods

The methods include database development and descriptive analysis of CCTs.

### Database development

**Fig. 1:**
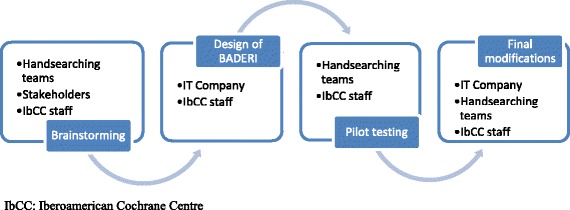
Flowchart for the development of BADERI: tasks and personnel responsible

The database development process started in September 2013. Through brainstorming and discussion among staff and stakeholders involved in handsearching projects, we identified needs that should be solved in order to ensure the viability of the handsearching enterprise. We contracted the services of an information technology (IT) company to set up a webpage that would host the proposed database, incorporating features that would address the identified needs. The development process of the database is summarized in Fig. 1.

We iteratively assessed and tested different versions of the database, proposing at each time changes and add-ons to improve its accessibility, practicality, and usefulness. We uploaded the titles of more than 1500 journals, which had been identified in a previous study [21], that were potentially eligible for handseaching. In order to be included in this database, journals had to publish original articles on biomedical research, regardless of language or country of origin and of whether they were indexed in any database or had an impact factor. Journals that focussed exclusively on academic or promotional activities were excluded. The entire development process lasted 18 months until the database was ready for pilot testing among different handsearching teams in March 2015.

### Pilot testing of the database

Three handsearching teams were recruited for pilot testing the database. They were involved in handsearching activities for Gynaecology, Ophthalmology, and Orthopaedics and Traumatology. They were asked to enter references of identified CCTs into the database, including study authors, journal of publication, and volume, year, and issue number where the CCT was published, among other data as described below. They were also required to verify that the data had been entered correctly using the built-in search engine and to create reports to track the progress of their handsearching activities.

### Database updating

Based on the feedback received from the staff that pilot tested the database, we incorporated new features and modified existing ones, once again through iterative discussion among stakeholders and staff at the IbCC. Additionally, it was planned that the design and features of the database will be revisited continuously as more users give us feedback about their experience in implementing their handsearching activities.

### Launching of the database and early activities

Once the final version of the database was approved, we proceeded to train the staff involved in handsearching projects. As reported elsewhere, the handsearching activities are conducted following the guidelines provided by the Cochrane Collaboration [1, 15]. These require that each journal issue be carefully inspected, assessing not only original articles but also editorials, letters to the editor, abstracts, and conference presentations. The recommended steps are (1) reading the table of contents, (2) locating keywords in the title of the article (e.g. randomized, random, blinded, etc.), (3) reading the abstract, and (4) reading the methods section in the full text of the article. Handsearching must be conducted retrospectively starting with the latest available issue of the corresponding journal. All personnel involved in handsearching are required to complete a pilot test, consisting of identifying CCTs in a volume of a journal that had previously been handsearched by personnel expert in the field.

### Eligibility criteria

In order to be eligible for inclusion in the database, studies had to meet the following criteria, as per the guidelines provided by the Cochrane collaboration to classify studies as CCTs: they had to (1) compare treatments in humans, (2) be prospective; interventions must have been planned before the study took place, (3) compare two or more interventions, one of which can be a no-treatment control group or a placebo, and (4) have a random or quasi-random method of allocation to treatment [1]. Random allocation was defined as the explicit adoption of random methods for assignment of participants to study arms, such as computer-generated random numbers. Quasi-random allocation was defined as the adoption of less stringent methods that can be used to generate comparable groups for the study arms, such as assignment by date of birth, even and odd numbers, or medical record number [28].

We included CCTs regardless of whether they were published as full-text articles or just in abstract format (such as in reports of conference proceedings). We excluded articles that were translations of studies published in other languages in order to avoid duplicates once these references are sent to CENTRAL.

### Data extraction

For each journal title included in BADERI, we recorded the following information: International Standard Serial Number (ISSN), country of origin, medical specialty, and years of publication. The database allows up to two medical specialties per journal. Each identified CCT was entered in the database and filed under the corresponding journal. We recorded the following information for each identified CCT: title in Spanish and English (if available); author(s), in a format compatible with the requirements of CENTRAL; year, volume, issue number, and pages of publication (when appropriate); and method of randomization. The name of the person who identified the CCT, the year when it was identified, and whether the corresponding full text is available were also recorded. There was a field where comments could be entered as needed. Lastly, we translated into English the titles of CCTs that were available only in Spanish.

### Data analysis

We used descriptive statistics to analyse the progress of the implementation of the database for different handsearching projects. This analysis was performed using reports generated by the database, which are exported in Excel® format, version 2010, Microsoft Office, Redmond, WA, USA. Additionally, all references entered in BADERI can be exported in ProCite format and submitted for inclusion in CENTRAL for their potential inclusion in systematic reviews and other documents of synthesis [22, 23].

## Results

### Database development (design and updating)

The proposed database was named BADERI (Database of Iberoamerican Clinical Trials and Journals, by its initials in Spanish). BADERI was officially launched in October 2015 and can be accessed at www.baderi.com/login.php (login and password needed, which can be generated upon request). BADERI is free of cost to all users.

After the brainstorming and discussion sessions, we agreed upon the features that were added to BADERI, the flow that users would follow to enter new references into the database, and the format of the reports. We subsequently assessed different versions of the database and approved the interface proposed by the IT company that set up BADERI.

Starting in March 2015 and during the next 3 months, BADERI was pilot tested. Three handsearching teams entered 203 references to CCTs, performed free text searches for a convenience sample of references to verify they had been correctly entered, and generated the available reports to track the work they had completed. This exercise allowed us to incorporate important changes to BADERI, the most relevant of which were (1) adding fields for recording additional information from identified CCTs (e.g. name of the person who had identified the CCT), (2) integrating drop-down menus for some fields (e.g. for method of randomization), (3) changing the location of links (e.g. links for generating a new journal or a new reference), and (4) suggesting formats or data to be included in reports (e.g. reports that could be filtered by journal, country, or medical specialty).

Since the database has been launched, we have received further feedback from users on how to improve the usability of BADERI. We update the database on a regular basis accordingly.

### Main characteristics

The login page prompts users to enter a username and password, which are assigned upon request. Users can be assigned to one or more handsearching projects (medical specialty), to handsearch journals from specific countries, and be granted different roles according to their responsibilities in the team. These roles include general administrator, or overall coordinator for all handsearching activities; local administrator, or coordinator at the country or medical specialty level; and reviewer (user/handsearcher), responsible for uploading references to identified CCTs. This distribution of tasks encourages teamwork while allowing team leaders to oversee the progress of the project.

Once signed in, users are prompted to a home page with general instructions on how to complete different tasks. The home page also includes acknowledgement to the entities that have financially supported BADERI as well as to the IT company responsible for its development. Lastly, users can find contact information for submitting inquiries or reporting issues with BADERI.

BADERI is divided into four subsections that can be accessed by clicking on different tabs in the home page: ‘Administration’, ‘References’, ‘Reports’, and ‘Search’.

The ‘Administration’ subsection allows one to assign each user different roles and handsearching projects, specifically per country and medical specialty. It also provides a list of all registered users. This subsection is centrally overseen from the IbCC headquarters in Barcelona, Spain, as well as by remotely situated local administrators.

Next, the ‘References’ subsection provides two subsections where references to published and non-published CCTs (i.e. grey literature) can be entered. The process to enter a new reference can be summarized as follows. First, users can determine, through a free text search in the upper section of the page, if the journal where the reference was published is already registered in BADERI. Users can choose to create a new journal title and enter all relevant information or to modify existing information. The same process is followed for selecting the year, volume, and issue of publication. Users can then click the ‘new article’ button within the corresponding issue, which prompts them to enter all information related to the CCT and its identification. BADERI automatically records the user person who entered this information, as well as the date and time.

The ‘Reports’ subsection allows spreadsheets to be exported in Excel format to monitor handsearching activities. The reports provide all available information for the journals and the CCTs registered in the database and can be filtered per journal(s), country(ies), or medical specialty(ies). There are three types of reports: handsearched journals, identified CCTs, and overall report of activities.

Lastly, the ‘Search’ subsection contains the search engine. This engine allows retrieving references through free text searches for journal title, author, or title of the article.

BADERI has a built-in feature, also available under the ‘Reports’ subsection, which allows the exportation of all bibliographic information available in the database in a ProCite file. This file can then be directly uploaded into CENTRAL. Additional file 1 provides screenshots of each of the four subsections in BADERI.

### Results of BADERI early activities

As of August 2016, a total of 6284 references to CCTs had been uploaded to BADERI. These references correspond to a variety of handsearching projects completed by members of the IbCN. Table 1 provides a sorting of these references per country of publication.

**Table 1:** CCTs in BADERI per country of publication

Country	No. of CCTs (*n*)	Percentage
Spain	4745	75.5
Chile	676	10.8
Mexico	249	4.0
Colombia	190	3.0
Cuba	183	2.9
Argentina	94	1.5
Peru	64	1.0
Uruguay	42	0.66
Ecuador	15	0.23
Venezuela	12	0.19
Guatemala	8	0.13
El Salvador	4	0.06
Paraguay	2	0.03
Total	6284	100.0

**Fig. 2:**
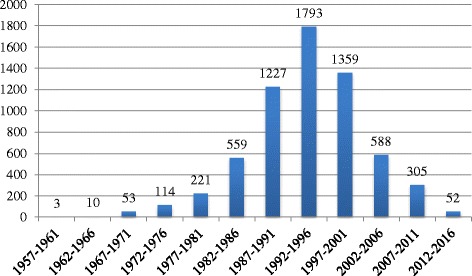
CCTs in BADERI published per 5-year period

The year of publication ranged between 1957 and 2016. The distribution of articles published per 5-year period reveals that most of the articles currently in BADERI were published between 1987 and 2001 (4379, 69.7%) (Fig. 2).

The identified CCTs were published in a total of 420 journals related to more than 46 medical specialties (Table 2).

**Table 2:** CCTs in BADERI per medical specialty

Medical specialty	No. of CCTs (*n*)	Percentage
General and Internal Medicine	915	14.5
Anaesthesiology	626	10.0
Cardiology	601	9.6
Gastroenterology	576	9.2
OB-GYN	372	5.9
Pulmunology	305	4.9
Paediatrics	287	4.6
Psychology	286	4.6
Oncology	264	4.2
Surgery (general)	188	3.0
Dentistry	153	2.4
Pharmacology	148	2.4
Immunology	139	2.2
Neurology	137	2.2
Nutrition	132	2.1
Dermatology	114	1.8
Public Health	111	1.8
All others	930	14.6
Total	6284	100.0

The most common journals and countries of precedence are listed in Table 3.

**Table 3:** CCTs in BADERI per journal of publication and country

Journal	Country	No. of CCTs (*n*)	Percentage
*Revista Española de Anestesiología y Reanimación*	Spain	438	7.0
*Gastroenterología y Hepatología*	Spain	388	6.2
*Archivos de Bronconeumología*	Spain	237	3.8
*Revista Española de Cardiología*	Spain	221	3.5
*Medicina Clínica*	Spain	177	2.7
*Medicina Intensiva*	Spain	154	2.5
*Revista Chilena de Anestesia*	Chile	140	2.2
*Revista Clínica Española*	Spain	124	2.0
*Hipertensión*	Spain	117	1.9
*Nutrición Hospitalaria*	Spain	113	1.8
*Revista Médica de Chile*	Chile	108	1.7
*Alergología et Immunopathologia*	Spain	97	1.5
*Annals de Medicina*	Spain	96	1.5
*Revista Española de Enfermedades Digestivas*	Spain	89	1.4
*Cirugía Española*	Spain	75	1.2
*Sangre*	Spain	68	1.1
*Neurología (Barcelona)*	Spain	66	1.1
*Progresos de Obstetricia y Ginecología*	Spain	64	1.0
*Atención Primaria*	Spain	62	1.0
All others		3450	54.9
Total		6284	100.0

Authors of a large proportion of the identified CCTs (3050, 48.4%) did not specifically describe the method they implemented for achieving randomization of participants. Among the remaining authors, 2105 (33.5%) implemented random allocation, whereas 1129 (18.1%) implemented quasi-random allocation. We found very few CCTs with titles translated into English; two professional translators recorded the corresponding information in BADERI. Lastly, a report of all the identified CCTs was generated in ProCite format and uploaded to CENTRAL.

## Discussion

We have developed BADERI, an online database to facilitate the management of handsearching of CCT projects. BADERI has several features that address the logistic challenges of this type of undertaking. Through a user-friendly interface, BADERI allows maintaining a repository of the journals that have been handsearched, the number of articles reviewed, and full bibliographic references to the identified CCTs. This information can be easily exported in Excel format for descriptive analyses or in ProCite format for its inclusion in CENTRAL. Users can be assigned to one or more handsearching projects (medical specialty or by country/ies) and be granted general administrator, local administrator (at the country level), or handsearching roles. This distribution of tasks encourages teamwork while allowing team leaders to oversee the progress of the project.

The development of BADERI relied on input from experts with first-hand knowledge of the challenges that handsearching projects entail. In addition, the database was pilot tested by personnel with ample experience completing handsearching projects. The involvement of these stakeholders guarantees that BADERI incorporates features and functions to expedite handsearching projects, while stimulating the participation of more volunteers working from remote locations. BADERI will also provide an entry door to people who may be interested in systematic reviews even before they receive the corresponding formal training by allowing them to participate in the identification of potentially eligible CCTs.

Most importantly, all the material contained in BADERI will be made available for its potential inclusion in systematic reviews and other documents of synthesis. BADERI contains all the information required by CENTRAL, and all data can be exported in ProCite format and uploaded directly into CENTRAL. Based on our previous experience, BADERI significantly simplifies the process of submitting data to CENTRAL. This year alone, we have been able to submit more than 3000 references, more than we had managed to in all previous years together, and we expect to send more than 2000 more within the upcoming semester.

To our knowledge, there are no other initiatives or studies that have addressed the challenges of handsearching projects. As reported by several other studies [29–32], BADERI can help overcome some of the shortcomings of literature searches by facilitating the implementation of handsearching initiatives on behalf of different institutions. BADERI will also give visibility to research published in non-indexed journals and journals published in the Spanish language by facilitating the inclusion of CCTs identified via handsearching into CENTRAL.

### Strengths and limitations

The main strengths of our study include the expertise in handsearching of the personnel involved in the design, setup, and pilot testing of BADERI. Furthermore, these personnel have ample experience in completing systematic reviews and in developing research projects in the methodology of systematic reviews and clinical practice guidelines. Additionally, the database we propose is free of cost and user-friendly, which facilitates its adoption among handsearching teams. Lastly, BADERI’s features to export data in spreadsheets and ProCite format are unique, streamlining the analysis of data registered in BADERI and its submission to CENTRAL.

Our work is subject to some limitations. BADERI may require input and pilot testing on behalf of other stakeholders, especially beyond projects conducted within the Cochrane Collaboration. Similarly, access to use BADERI is granted upon request to a local or general administrator, which may be a barrier to its uptake. However, we are committed to collaborating with any entity interested in learning how to use BADERI to meet their specific needs and to granting access to the database and its contents to any interested party.

Given that we are still in the process of uploading identified CCTs into BADERI, we cannot draw firm conclusions about the main characteristics of this body of work. We hope that as we complete the process of compiling in BADERI the results of all handsearching activities conducted so far, we will be better able to conduct descriptive studies regarding the features of these studies.

### Implications for practice

The BADERI database will serve handsearching teams across several countries and institutions. All personnel involved in these activities will be granted access to BADERI free of charge. Furthermore, BADERI could be easily adapted to fulfil the handsearching management needs of other entities. BADERI can also be a useful resource for other initiatives or groups that need to register and monitor existing journals and corresponding handsearching activities.

We are currently providing training to several handsearching teams from Spain and Latin America on how to report the results of their work via BADERI. As they implement BADERI in their handsearching activities, they provide feedback that helps us to adjust and improve the database. We are also finishing the upload of handsearching projects that were conducted in the past but that have not been added to BADERI. Once this process is complete, this material will be readily available for consultation.

### Implications for research

The handsearching of CCTs is a crucial complement to electronic searches in order to identify all the available evidence that can potentially be used in systematic reviews and other documents of synthesis. The IbCC has made it a top priority to foster current and future handsearching projects of biomedical journals in different medical fields, for which the BADERI database will be an invaluable aid. We are currently finishing the upload of references identified in previous handsearching projects and its submission to CENTRAL. There is also a need to critically assess the results and quality of the CCTs included in BADERI.

## Conclusions

BADERI allows the efficient management of handsearching activities across different countries and institutions. References to all CCTs available in BADERI can be readily submitted to CENTRAL for their potential inclusion in systematic reviews. There is a need to critically assess the main characteristics and methodological quality of the CCTs included in BADERI.

## Additional file

Additional file 1: BADERI subsections. (PDF 541 kb)
