# Supplementary material for: BADERI: an online database to coordinate handsearching activities of controlled clinical trials for their potential inclusion in systematic reviews
Source: Trials. 2017 Jun 13;18:273. doi: 10.1186/s13063-017-2023-3 (PMC5470310; doi:10.1186/s13063-017-2023-3)

Additional file 1: BADERI Subections

http://baderi.com/login.php

Welcome page

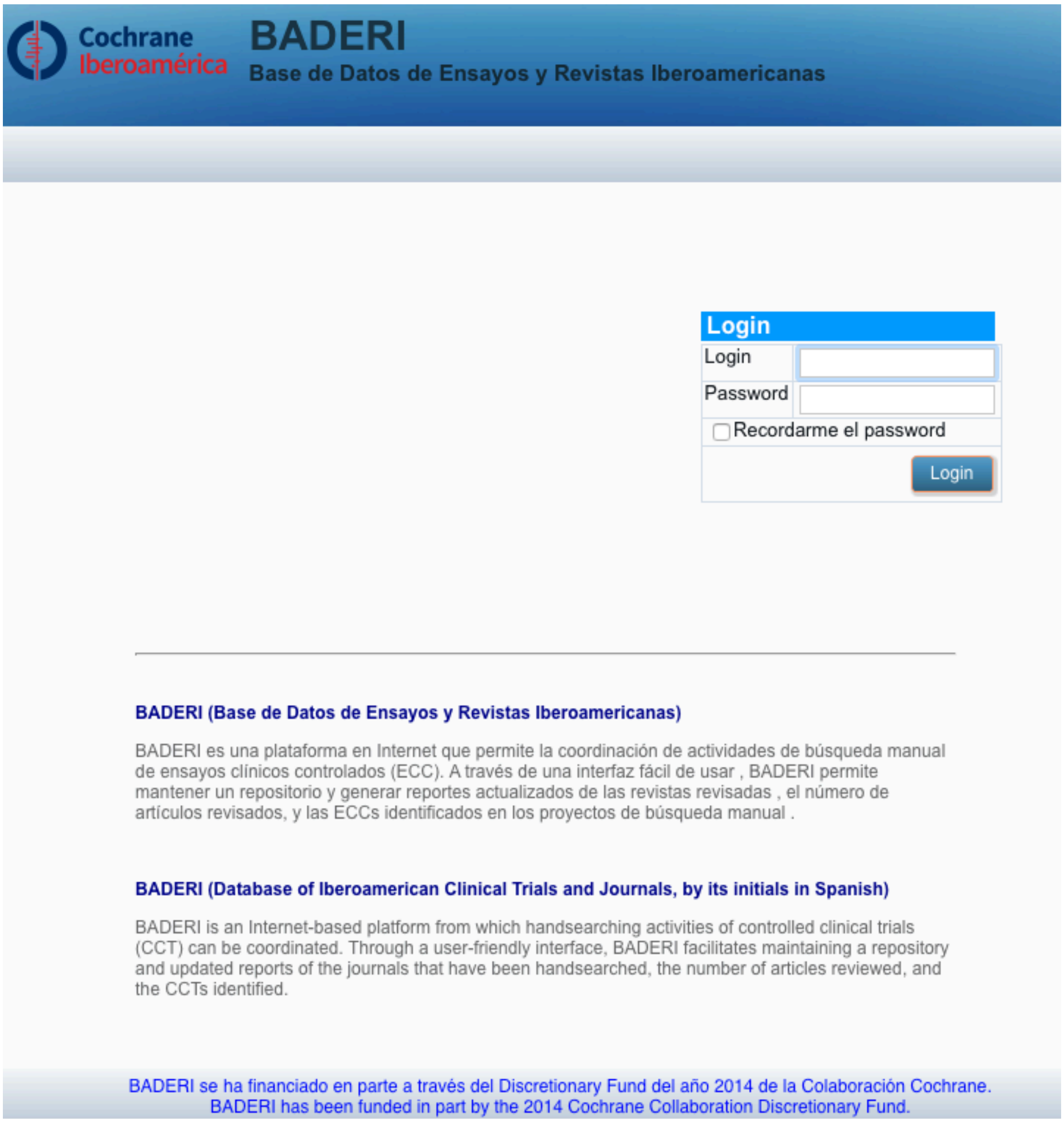

Administration tab

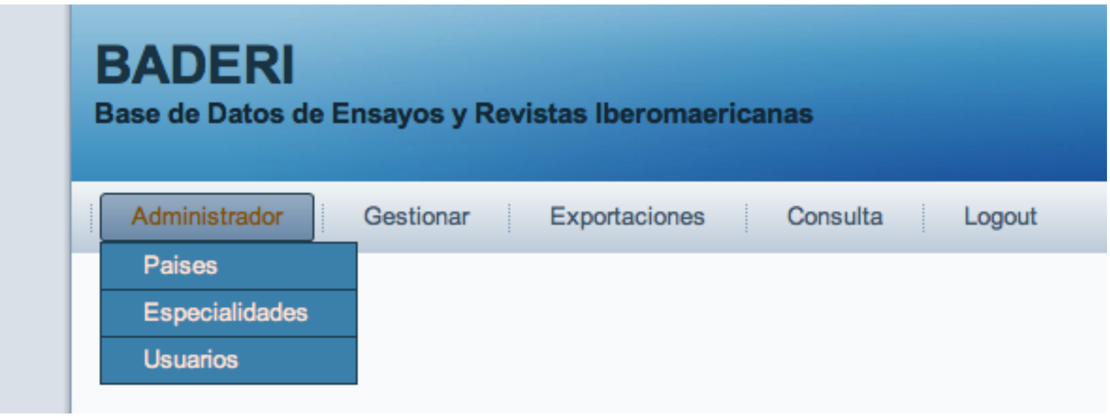

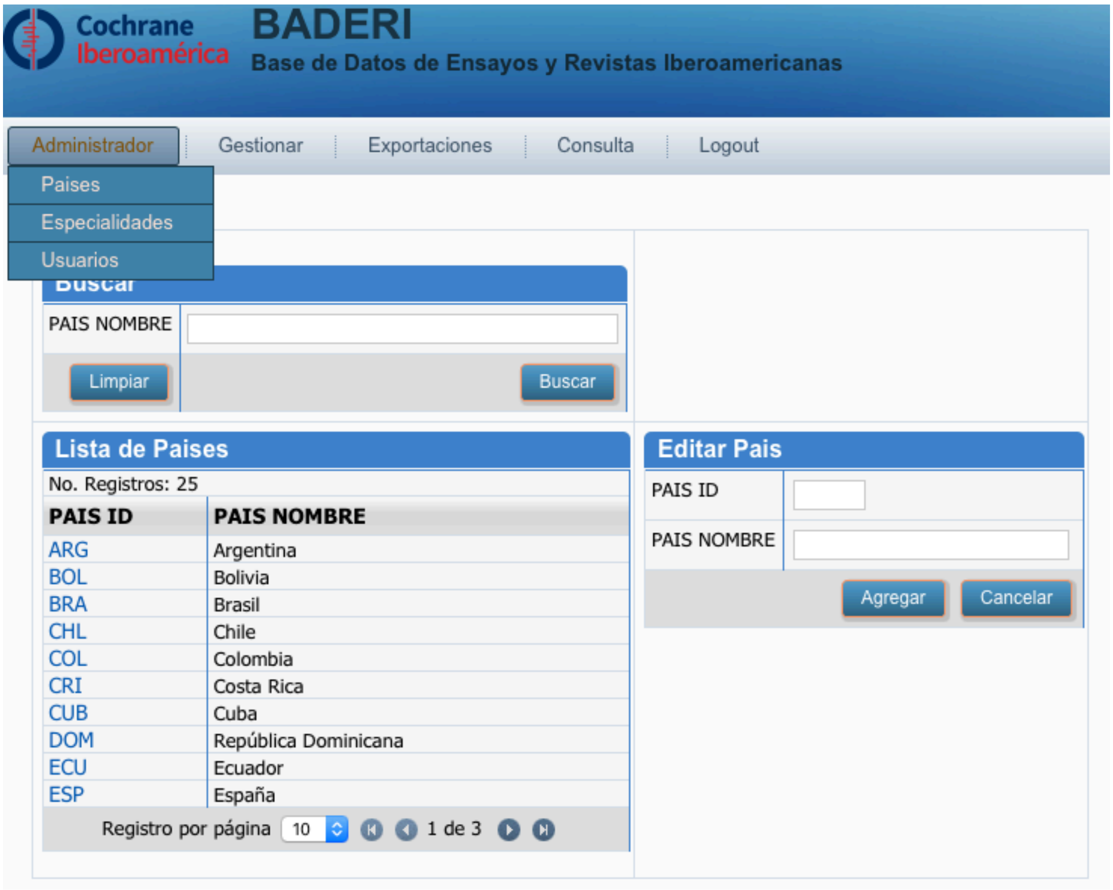

References tab

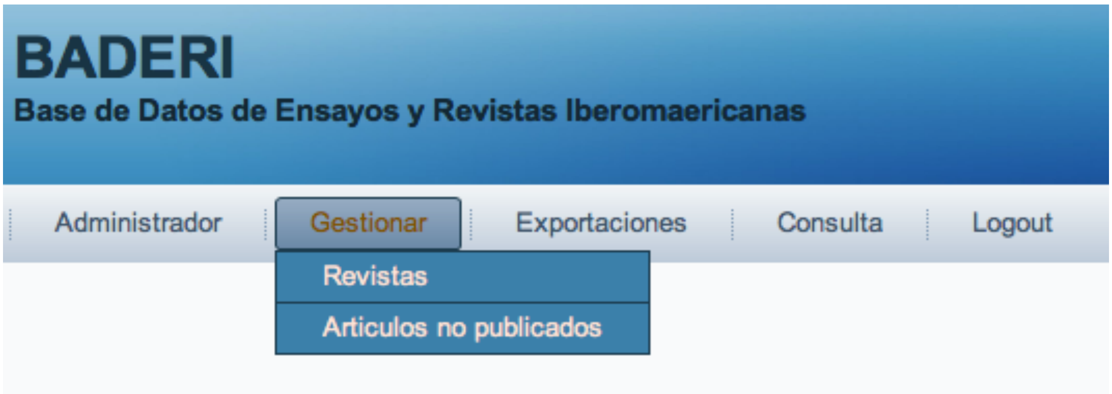

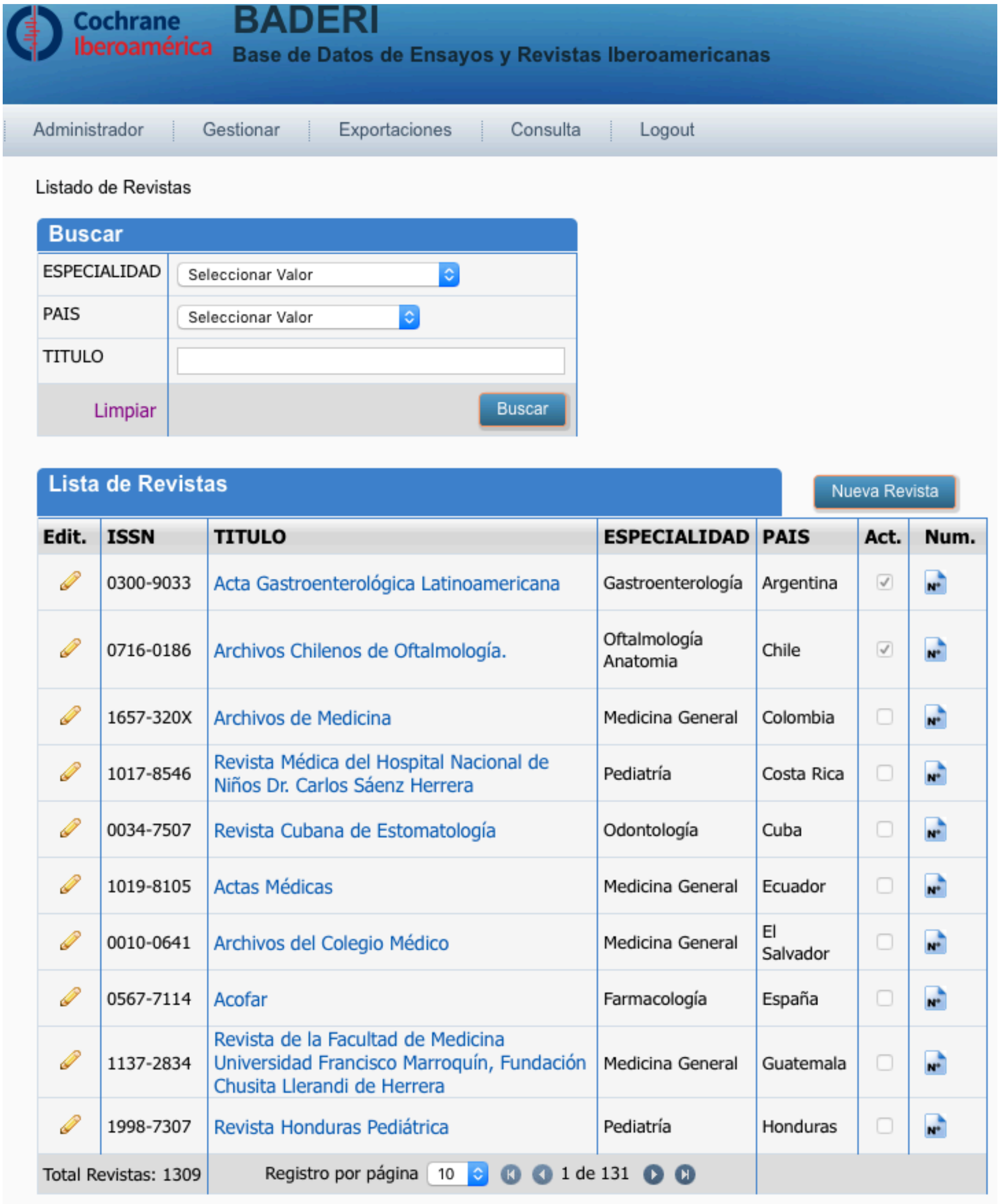

Edit.	ISSN	TITULO	ESPECIALIDAD	PAIS	Act.	Num.
	0300-9033	Acta Gastroenterológica Latinoamericana	Gastroenterología	Argentina	☑	
	0716-0186	Archivos Chilenos de Oftalmología.	Oftalmología Anatomia	Chile	☑	
	1657-320X	Archivos de Medicina	Medicina General	Colombia	☐	
	1017-8546	Revista Médica del Hospital Nacional de Niños Dr. Carlos Sáenz Herrera	Pediatría	Costa Rica	☐	
	0034-7507	Revista Cubana de Estomatología	Odontología	Cuba	☐	
	1019-8105	Actas Médicas	Medicina General	Ecuador	☐	
	0010-0641	Archivos del Colegio Médico	Medicina General	El Salvador	☐	
	0567-7114	Acofar	Farmacología	España	☐	
	1137-2834	Revista de la Facultad de Medicina Universidad Francisco Marroquín, Fundación Chusita Llerandi de Herrera	Medicina General	Guatemala	☐	
	1998-7307	Revista Honduras Pediátrica	Pediatría	Honduras	☐	

Total Revistas: 1309 Registro por página 10 1 de 131

BADERI reports by journal, issue numbers, references to articles, overall report, and articles not published in any journal

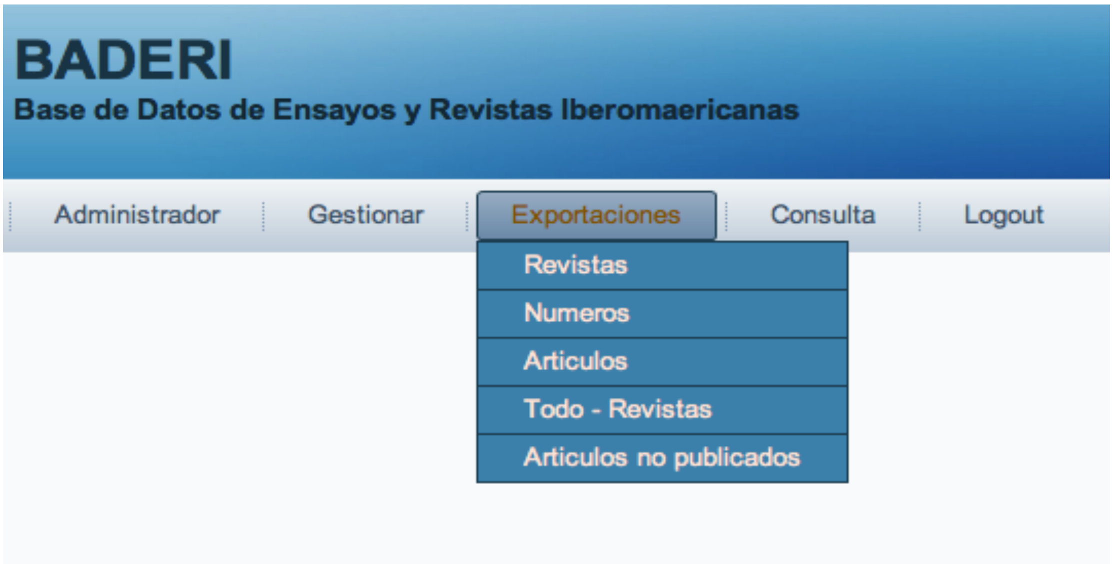

Search engine of controlled clinical trials by free text

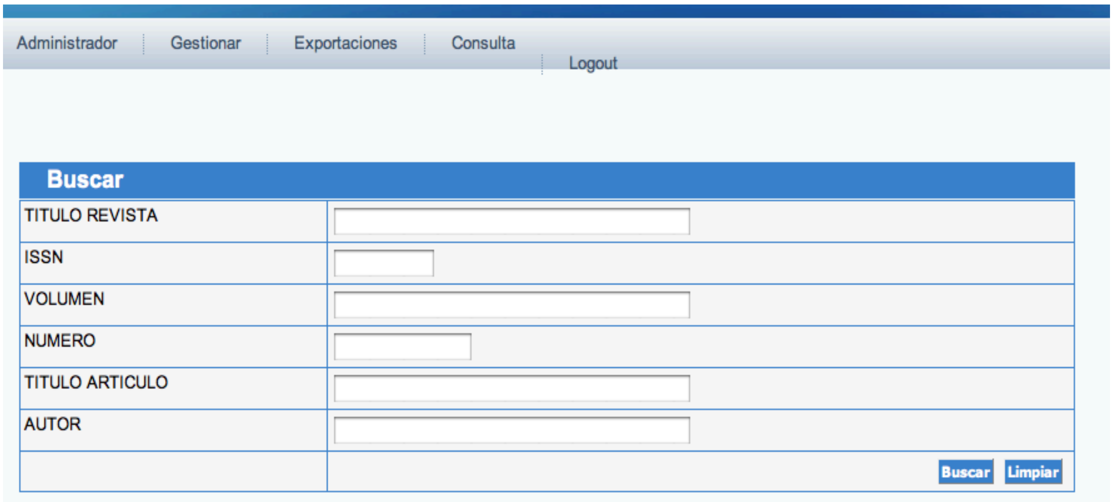

Supplement: Additional file 1: — BADERI subsections. (PDF 541 kb) [file 13063_2017_2023_MOESM1_ESM.pdf]
